# Spread of Plague by Respiratory Droplets or Ectoparasites

**DOI:** 10.3201/eid2405.172067

**Published:** 2018-05

**Authors:** Charles Morris Evans

**Affiliations:** University of Birmingham, Birmingham, UK

**Keywords:** plague, pneumonic plague, ectoparasites, epidemic, parasites

**To the Editor:** Drancourt and Raoult (*1*) have emphasized the risk of overestimation of pneumonic plague contagion by respiratory droplets and hypothesize that only transmission of Yersinia* pestis* by ectoparasites, such as lice and fleas, by close contact with infected humans can sustain outbreaks and epidemics. The outbreak of pneumonic plague in Madagascar in 2017 (*2*) reminds us that plague remains a potential serious threat in locations that are relatively inaccessible or have limited capacity for a robust public health response. Records describe substantial outbreaks of pneumonic plague (*3*) but portray a more dangerous disease than that described by Drancort and Raoult. High rates of transmission are possible (*4*) when pneumonic plague is spreading through social networks, in a way similar to that observed in West Africa during the recent epidemic of Ebola virus disease (*5*). The Ebola virus is not thought to be easily transmitted but is clearly capable of generating a sustained epidemic.

The role of ectoparasites in the transmission of *Y. pestis* should not be dismissed. However, until a substantial epidemic has been documented with this proven etiology, this explanation of plagues, both historical and modern, must remain in the realm of conjecture.

## References

[1] Drancourt M, Raoult D. Investigation of pneumonic plague, Madagascar. Emerg Infect Dis. 2018;24:183. 10.3201/eid2401.170760 29260670 PMC5749468

[2] World Health Organization. Plague outbreak Madagascar. External situation report 11. 2017 Nov 17 [cited 2018 Mar 6]. http://apps.who.int/iris/bitstream/10665/259479/1/Ex-PlagueMadagascar17112017.pdf

[3] Teh WL. The second pneumonic plague epidemic in Manchuria, 1920–21: I. A general survey of the outbreak and its course. J Hyg (Lond). 1923;21:262–88. 10.1017/S0022172400031508 20474780 PMC2167333

[4] Evans CM, Egan JR, Hall I. Pneumonic plague in Johannesburg, South Africa, 1904. Emerg Infect Dis. 2018;24:95–102. 10.3201/eid2401.161817

[5] Faye O, Boëlle PY, Heleze E, Faye O, Loucoubar C, Magassouba N, et al. Chains of transmission and control of Ebola virus disease in Conakry, Guinea, in 2014: an observational study. Lancet Infect Dis. 2015;15:320–6. 10.1016/S1473-3099(14)71075-8 25619149 PMC4373532

